# A Therapeutic Relational Agent for Reducing Problematic Substance Use (Woebot): Development and Usability Study

**DOI:** 10.2196/24850

**Published:** 2021-03-23

**Authors:** Judith J Prochaska, Erin A Vogel, Amy Chieng, Matthew Kendra, Michael Baiocchi, Sarah Pajarito, Athena Robinson

**Affiliations:** 1 Stanford Prevention Research Center School of Medicine Stanford University Stanford, CA United States; 2 Department of Psychiatry & Behavioral Sciences School of Medicine Stanford University Stanford, CA United States; 3 Department of Epidemiology & Population Health School of Medicine Stanford University Stanford, CA United States; 4 Woebot Health San Francisco, CA United States

**Keywords:** artificial intelligence, conversational agent, chatbot, addiction, substance misuse, treatment, acceptability, feasibility, craving, psychoeducation, psychotherapeutic, mobile phone

## Abstract

**Background:**

Misuse of substances is common, can be serious and costly to society, and often goes untreated due to barriers to accessing care. Woebot is a mental health digital solution informed by cognitive behavioral therapy and built upon an artificial intelligence–driven platform to deliver tailored content to users. In a previous 2-week randomized controlled trial, Woebot alleviated depressive symptoms.

**Objective:**

This study aims to adapt Woebot for the treatment of substance use disorders (W-SUDs) and examine its feasibility, acceptability, and preliminary efficacy.

**Methods:**

American adults (aged 18-65 years) who screened positive for substance misuse without major health contraindications were recruited from online sources and flyers and enrolled between March 27 and May 6, 2020. In a single-group pre/postdesign, all participants received W-SUDs for 8 weeks. W-SUDs provided mood, craving, and pain tracking and modules (psychoeducational lessons and psychotherapeutic tools) using elements of dialectical behavior therapy and motivational interviewing. Paired samples t tests and McNemar nonparametric tests were used to examine within-subject changes from pre- to posttreatment on measures of substance use, confidence, cravings, mood, and pain.

**Results:**

The sample (N=101) had a mean age of 36.8 years (SD 10.0), and 75.2% (76/101) of the participants were female, 78.2% (79/101) were non-Hispanic White, and 72.3% (73/101) were employed. Participants’ W-SUDs use averaged 15.7 (SD 14.2) days, 12.1 (SD 8.3) modules, and 600.7 (SD 556.5) sent messages. About 94% (562/598) of all completed psychoeducational lessons were rated positively. From treatment start to end, in-app craving ratings were reduced by half (87/101, 86.1% reporting cravings in the app; odds ratio 0.48, 95% CI 0.32-0.73). Posttreatment assessment completion was 50.5% (51/101), with better retention among those who initially screened higher on substance misuse. From pre- to posttreatment, confidence to resist urges to use substances significantly increased (mean score change +16.9, SD 21.4; *P*<.001), whereas past month substance use occasions (mean change −9.3, SD 14.1; *P*<.001) and scores on the Alcohol Use Disorders Identification Test-Concise (mean change −1.3, SD 2.6; *P*<.001), 10-item Drug Abuse Screening Test (mean change −1.2, SD 2.0; *P*<.001), Patient Health Questionnaire-8 item (mean change 2.1, SD 5.2; *P*=.005), Generalized Anxiety Disorder-7 (mean change −2.3, SD 4.7; *P*=.001), and cravings scale (68.6% vs 47.1% moderate to extreme; *P*=.01) significantly decreased. Most participants would recommend W-SUDs to a friend (39/51, 76%) and reported receiving the service they desired (41/51, 80%). Fewer felt W-SUDs met most or all of their needs (22/51, 43%).

**Conclusions:**

W-SUDs was feasible to deliver, engaging, and acceptable and was associated with significant improvements in substance use, confidence, cravings, depression, and anxiety. Study attrition was high. Future research will evaluate W-SUDs in a randomized controlled trial with a more diverse sample and with the use of greater study retention strategies.

**Trial Registration:**

ClinicalTrials.gov NCT04096001; http://clinicaltrials.gov/ct2/show/NCT04096001.

## Introduction

Misuse of substances is common, can be serious and costly to society, and often goes untreated due to barriers to accessing care. Globally, 3.5 million people die from alcohol and illicit drug use each year [1]. The disease burden of alcohol and illicit drug addiction is the highest in the United States [2]. Over 20 million Americans (aged 12 years and older) had a substance use disorder (SUD) in 2018, 73% had an alcohol use disorder, 40% had an illicit drug use disorder, and 13% had both alcohol and illicit drug use disorders [3]. Approximately half (47%) of Americans with an SUD had a co-occurring mental illness. Treatment of depression and anxiety, the most common psychiatric comorbidities among patients with SUDs, may reduce craving and substance use and enhance overall outcomes [4].

In 2018, less than 1 in 5 individuals with a SUD received addiction treatment [3]. Alcohol and illicit drug misuse and addiction cost the United States over US $440 billion annually in lost workplace productivity, health care expenses, and crime-related costs [5]. Potential effects on individuals include an array of physical and mental health problems, overdose, trauma, and violence [5].

Web-based interventions and digital health apps may reduce or eliminate common, significant barriers to traditional SUD treatment (eg, stigma; financial, time, and transportation constraints; lack of access to qualified providers; challenges navigating complex treatment systems; and low perceived utility) [6]. Preliminary evidence suggests that digital SUD interventions affect substance use behavior [6,7] and have the potential to reduce the population burden of SUDs. To date, most digital SUD interventions have been delivered on a web platform, rather than via mobile apps. The widespread use of smartphones makes app-based intervention delivery a viable and scalable medium. In 2019, about 8 out of 10 White, Black, and Latinx adults owned a smartphone [8]. Although lower-income adults were less likely to own a smartphone than higher-income adults, they were more likely to rely on smartphones for internet access [9]. In a 2015 survey, 58% of mobile phone owners reported downloading a health app [10]. Texting is the most widely and frequently used app on a smartphone, with 97% of Americans texting at least once a day [11].

Automated conversational agents can deliver a coach-like or sponsor-like experience and yet do not require human implementation assistance for in-the-moment treatment delivery. As recent meta-analytic work suggests, conversational text-based agents may increase engagement and enjoyment in digitized mental health care [12], whereas most general mental health care apps face difficulty sustaining engagement with high dropout [13,14]. Conversational agents can provide real-time support to address substance use urges, unlike traditional in-person frameworks of weekly visits. The scale potential of conversational agents is unconstrained, immediate, and available to users in an instant [12]. Being nonhuman based also reduces perceived stigma. A study found that people were significantly more likely to disclose personal information to artificial intelligence when they believed it was computer- rather than human-monitored [15]. Users can develop a strong therapeutic alliance in the absence of face-to-face contact [16], even with a nonhuman app [17]. Digital environments can promote honest disclosure due to greater ease of processing thoughts [16] and reduced risk of embarrassment [17]. Finally, although conversational agents can present in different modalities, including text, verbal [18,19], and animation [20-25], preliminary research on modality for psychoeducation delivery specifically found that text-based presentation resulted in higher program adherence than verbal presentation [26].

Evidence for conversational agent interventions for addressing mental health problems is growing quickly and appears promising with regard to acceptability and efficacy [27]. Developed as a mental health digital app, Woebot is a text-based conversational agent available to check in with users whenever they have smartphone access. Using conversational tones, Woebot is designed to encourage mood tracking and to deliver general psychoeducation as well as tailored empathy, cognitive behavioral therapy (CBT)–based behavior change tools, and behavioral pattern insight. Among a sample of adults (N=70) randomly assigned to Woebot or an information only control group, Woebot users had statistically and clinically significant reductions in depressive symptoms (*F*_1,48_=6.03; *P*=.02) after 2 weeks of use, whereas those in the control group did not. Engagement with the app was high (averaging 12 interactions within 14 days) [18].

However, the efficacy of conversational agents for treating SUDs remains unknown. Woebot’s app-based platform and user-centered design philosophy make it a promising modality for SUD treatment delivery; it offers immediate, evidence-based tailored support in the peak moment of craving. An informal poll of Woebot users (in July 2018) indicated that 63% had interest in content addressing SUDs; 22% of surveyed users reported having 5 or more alcoholic drinks in a row within a couple of hours (ie, binge use) [28], and 5% endorsed using nonprescription drugs.

Although the efficacy of automated conversational agent digital therapeutics for SUDs is still untested, such products are commercially available, and few consumers are aware that the products lack evidence [29]. This study aims to adapt the original Woebot for the treatment of SUDs (W-SUDs), and test the feasibility, acceptability, and preliminary efficacy in a single-group pre-/posttreatment design.

## Methods

### Study Design

In a single-group design, we examined within-subject changes in self-reported substance use behavior, cravings, confidence to resist urges to use substances, mood symptoms (depression, anxiety), and pain from pre- to posttreatment. Intervention engagement data were collected from the Woebot app during the 8-week treatment period. Acceptability ratings were collected within the app and within the posttreatment survey. The study procedures were approved by the Institutional Review Board of Stanford Medicine.

### Sample Recruitment

Participants were recruited via the Woebot app, social media (eg, Facebook and Nextdoor), Craigslist, and Stanford staff and student wellness listservs. In addition, study flyers were posted in the San Francisco Bay Area, and email invitations were sent to participants from previous studies. Recruitment materials included the URL on a webpage describing the study for people with substance use concerns. Informed consent was required to screen for eligibility. Those who screened as eligible were asked to provide informed consent for participation in the study.

Inclusion criteria were all genders, aged 18 years to 65 years, residing in the United States, screening positive on the 4-item Cut down, Annoyed, Guilty, Eye opener-Adapted to Include Drugs (CAGE-AID) [30] (ie, score of 2 or higher), owning a smartphone for accessing Woebot, available for the 8-week study, willing to provide an email address, and English literate. The CAGE-AID has demonstrated validity, with high internal consistency in screening for problematic drug and alcohol use; a cutoff point of 2+ on the CAGE-AID has a sensitivity of 70% and specificity of 85% for identifying individuals with SUDs [30]. Study exclusion criteria were current pregnancy, history of severe alcohol or drug-related medical problems (eg, delirium tremens, seizure, liver disease, and hallucinations), opioid overdose requiring Narcan (naloxone), current opioid misuse without medication-assisted treatment, or attempted suicide within the past year.

**Figure 1:**
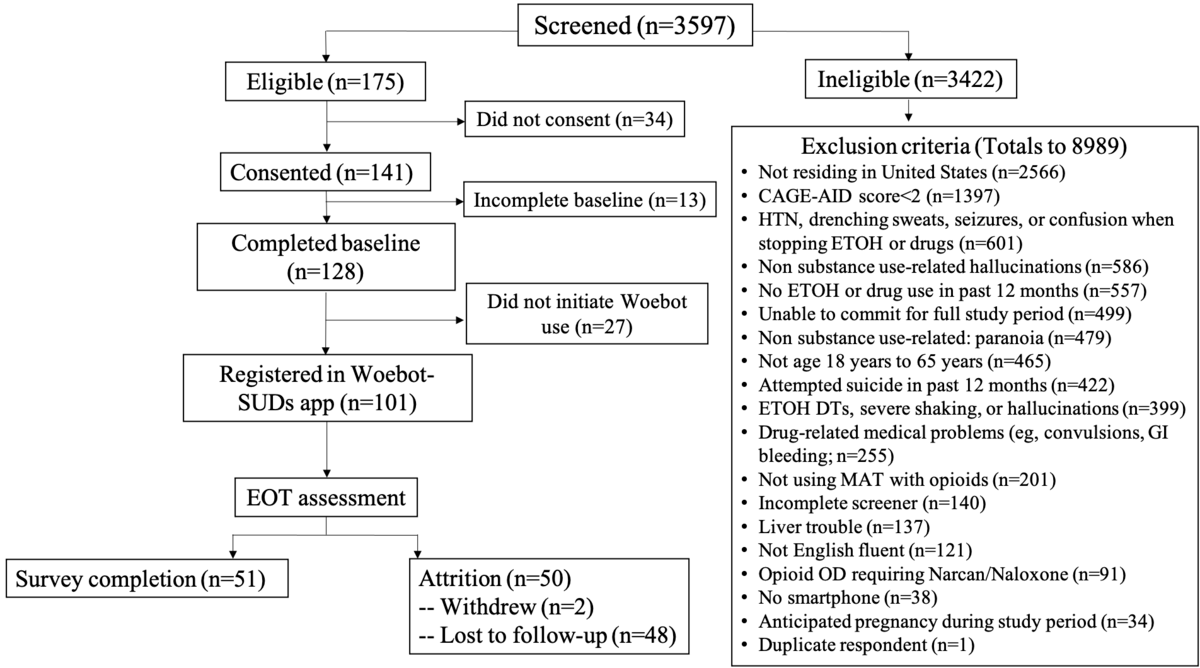
Study consort diagram. CAGE-AID: Cut down, Annoyed, Guilty, Eye Opener-Adapted to Include Drugs; DTs: delirium tremens; EOT: end of treatment; ETOH: ethyl alcohol; HTN: hypertension; MAT: medication-assisted treatment; OD: overdose; Woebot-SUDs: Woebot for the treatment of substance use disorders.

For this study, the target sample size was 50 participants; however, due to a high level of response and efficiency, enrollment was more than double our recruitment goal. Between March 27, 2020 and May 6, 2020, 3597 individuals were screened for study participation, with 3422 ineligible and 175 eligible individuals. Figure 1 shows the reasons for study exclusion, most frequently residing outside of the United States (2566/3433, 74.75%) and endorsing fewer than 2 criteria on the CAGE-AID (1397/3433, 40.69%). Of the 175 eligible participants, 141 provided informed consent to participate in the study, of whom 128 completed the baseline survey. The analytic sample consisted of 101 participants who ultimately registered with W-SUDs and initiated use. Among the 101 participants enrolled, 11 (10.9%) reported previous use of the Woebot app.

### Procedures

Those who provided informed consent and enrolled were asked to use W-SUDs for 8 weeks. Assessments were administered via Qualtrics at the beginning and end of the 8-week treatment period. Participants received a US $25 Amazon gift card at the end of the study for completing the posttreatment assessment.

### W-SUDs Intervention

Described in detail previously [18], Woebot is an automated conversational agent that delivers CBT in the format of brief, daily text-based conversations. The Woebot program is deployed through its own native apps on both iPhone and Android smartphones or devices. The app onboarding process introduces the automated conversational agent, explains the intended use of the device, how data are treated, and the limitations of the service (eg, it is not a crisis service). The user experience is centered around mood tracking and goal-oriented, tailored conversations that can, depending on user input and choice, focus on CBT psychoeducation, application of psychotherapeutic skills for change (eg, thought-challenging), mindfulness exercises, gratitude journaling, and/or reflecting upon patterns and lessons already covered. Each interaction begins with a general inquiry about context (eg, “What’s going on in your world right now?”) and mood (eg, “How are you feeling?”) to ascertain affect in the moment. Additional therapeutic process-oriented features of Woebot include delivery of empathic responses with tailoring to users’ stated mood(s), goal setting with regular check-ins for maintaining accountability, a focus on motivation and engagement, and individualized weekly reports to foster reflection. Users become familiar with Woebot, which is a friendly, helpful character that is explicitly not a human or a therapist but rather a guided self-help coach. Daily push notifications prompt users to check in.

**Figure 2:**
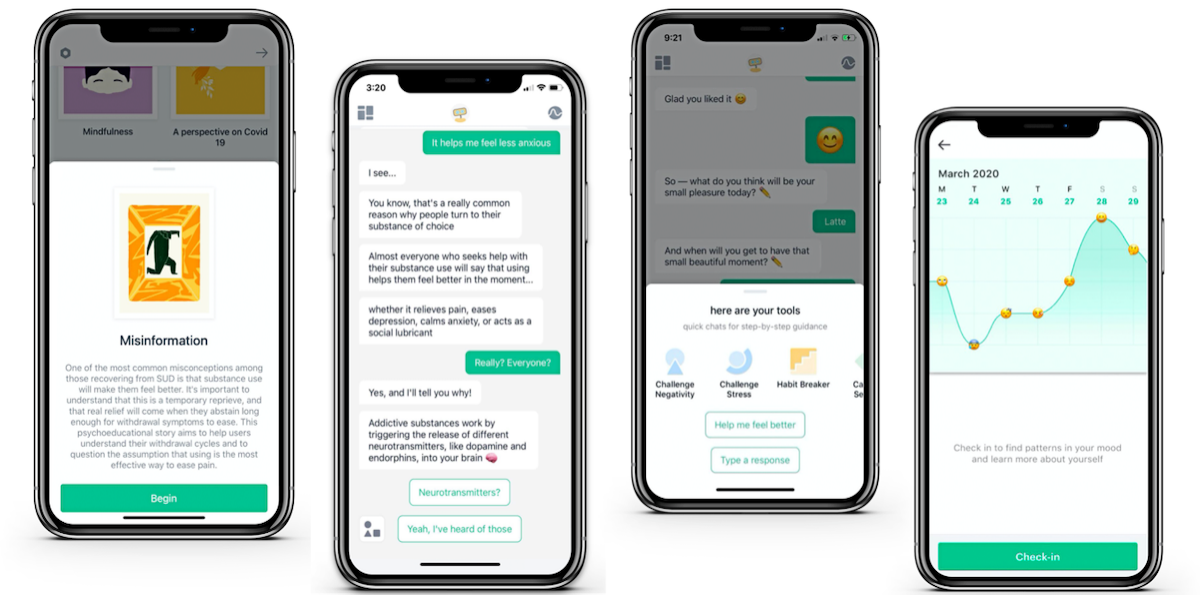
Sample screenshots of the Woebot for substance use disorders app: a psychoeducational lesson called Misinformation, the core conversational panel (featuring the Lesson Misinformation), and psychotherapeutic skills for behavior change and mood tracking.

We adapted W-SUDs, drawing upon motivational interviewing principles, mindfulness training, dialectical behavior therapy, and CBT for relapse prevention. Sample screenshots from the W-SUDs app are shown in Figure 2. In total, the W-SUDs intervention was developed as an 8-week program with tracking of mood, substance use craving, and pain, with over 50 psychoeducational lessons and psychotherapeutic skills.

CBT evidence-based, guided self-help treatments have ranged in length from 2 to 12 weeks [31-34], and the National Institutes for Clinical Excellence describes guided self-help as including 6 to 8 face-to-face sessions [35]. Early responsiveness to SUD treatment is predictive of long-term outcomes [36], and brief addiction treatments are efficacious [37]. Brief intervention can minimize potential dropout, a problem common to SUD treatment; [38] therefore, we designed W-SUDs as an 8-week treatment.

Woebot is not designed to address active suicidal ideation or overdose, and this was stated in the study informed consent. In addition, Woebot conversationally informs first-time users that it is not a crisis service. Woebot also has safety net detection that uses natural language processing algorithms to detect and flag several hundred possible harm-to-self phrases (including some misspellings and slang phrases) with 98% accuracy (sensitivity=97 and specificity=99; Woebot Health, unpublished data, September 2020). Woebot detects crisis language (eg, “want to cut myself”) and asks to confirm it with the user. If the user confirms, Woebot offers resources (eg, 9-1-1, suicide crisis hotlines), carefully curated with expert consultation. Woebot data indicate that users do not use Woebot for crisis management; approximately 6.3% trigger the safety net protocol, with 27% of those confirming that it is indeed a crisis when Woebot asks to confirm (ie, the true positive rate).

### Assessments

Demographic items were assessed at pretreatment; substance use, mental health, and pain measures were administered at pre- and posttreatment; serious adverse events and W-SUDs feasibility and acceptability were assessed at posttreatment; and W-SUDs use data were collected via the Woebot app over the 8-week intervention. Demographic items included self-reported sex, race and ethnicity, age, marital status, employment status, residential zip code, and sheltering-in-place status given the COVID-19 pandemic.

The Alcohol Use Disorders Identification Test-Concise (AUDIT-C), a widely used 3-item self-report measure based on the 10-item original AUDIT [39], assessed hazardous or harmful alcohol consumption in the past 3 months. A score of 4+ for men and 3+ for women indicated significant problems with alcohol consumption. The AUDIT-C has been found to be a valid screening test for heavy drinking and/or active alcohol abuse or dependence [39]. The Drug Abuse Screening Test-10 (DAST-10), a 10-item self-report measure adapted from the 28-item DAST [40], assessed consequences related to drug abuse, excluding alcohol and tobacco in the past 3 months. The last item of the DAST-10 regarding medical problems resulting from drug use was not reassessed because it was an exclusion criterion in the study screener; hence, the total possible range for the sample was 0-9, not 0-10. Total scores of 3+ indicated significant problems related to drug abuse. The DAST-10 has moderate test-retest reliability, sensitivity, and specificity [40]. For the AUDIT-C and DAST-10 measures at posttreatment, the reference period was the past 2 months, to reflect the period of intervention. Craving was assessed with a single item asking, “In the past 7 days, how much were you bothered by cravings or urges to drink alcohol or use drugs?”, with response options of not at all (0), a little bit (1), moderately (2), quite a bit (3), and extremely (4). The Brief Situational Confidence Questionnaire [41], a state-dependent measure, assessed self-confidence to resist the urge “right now” to drink heavily (self-defined) or use drugs in different situations reported on visual analog scales (100 mm lines) anchored from 0% “not at all confident” to 100% “totally confident.”

The Patient Health Questionnaire-8 item (PHQ-8), an 8-item scale, assessed depressive symptoms [42], and the Generalized Anxiety Disorder-7 item (GAD-7), a 7-item scale, assessed symptoms of generalized anxiety disorder [43]. Both the PHQ-8 and GAD-7 have good internal consistency and demonstrated convergent validity with measures of depression, stress, and anxiety. A total of 2 items assessed the history of therapy (ever and current) for mental health or substance use concerns. Lifetime psychiatric diagnoses were assessed using 10 items plus a write-in option for others. A single item assessed currently taking prescribed medications for a psychiatric diagnosis.

The treatment feasibility and acceptability of W-SUDs were assessed posttreatment using the Usage Rating Profile-Intervention (URP-I) Feasibility (6 items) and Acceptability (6 items) scales [44], the 8-item Client Satisfaction Questionnaire-8 questions (CSQ-8) [45], and the 12-item Working Alliance Inventory-Short Revised (WAI-SR) [46]. The URP-I item response options ranged from strongly disagree to strongly agree; the items were summed for a total score within each scale, with one feasibility item reverse coded. The CSQ-8 items have 4-point rating scales with response descriptors that vary. Internal consistency exceeds 0.90, and the total sum score ranges from 8 to 32, with higher total scores indicating higher satisfaction. The WAI-SR has three 4-item subscales, with 5-point rating scales, that reflect development of an affective bond in treatment and level of agreement with treatment goals and treatment tasks. Serious adverse events occurring in the 8 weeks after the start of the study were assessed for hospitalization related to substance use, suicide attempt, alcohol or drug overdose, and severe withdrawal (eg, delirium tremens). Positive endorsements were followed up with questions about the timing, diagnosis, and resolution. If additional details were needed to determine whether the event was study related, a team member reached out to the participant. Serious adverse events were reported to the study’s Data Safety Monitoring Board (DSMB) within 72 hours of the team learning of the event.

Participants’ W-SUDs app use, including days of app use, number of check-ins, and number of messages sent, was collected via the Woebot app, as were module completion rates, lesson acceptability ratings indicated on a binary scale (ie, a thumbs up or thumbs down emoticon), and mood impact after tools utilization (ie, feeling same, better, or worse after completion). In addition, on a daily basis, the W-SUDs app assessed mood, cravings or urges to use, and pain. In-the-moment emotional state was reported through emoji selection with a default menu of 19 total moods, including options for negative (angry, sad, and anxious), positive (happy and content), and average mood (okay), with an additional ability to type in free text emotion words and/or self-selected emoji expressions. Cravings were assessed as not at all (0), a little bit (1), moderately (2), quite a bit (3), or extremely (4). Physical pain was rated on a scale of 0 to 10.

### Data Analyses

Descriptive statistics (means and frequencies) were used to describe the sample and examine the ratings of program feasibility and acceptability. Paired samples *t* tests and McNemar nonparametric tests examined within-subject changes from pre- to posttreatment on measures of substance use, confidence, cravings, mood, and pain. Change scores were calculated (pre- minus posttreatment), and bivariate correlations were used to examine associations between changes in AUDIT-C and DAST-10 scores and changes in use occasions, confidence, and depression and anxiety scores. *t* tests were conducted to examine changes from pre- to posttreatment in substance use, confidence, mood, and pain by whether participants were currently in therapy or taking psychiatric medications. Posttreatment survey completion was 50.5% (51/101), with better retention among those with a higher CAGE-AID score at screening (γ=0.37; *P*=.02). Retention was lowest among those with a CAGE-AID score of 2 (7/26, 27%) and higher for those scoring 3 (22/38, 58%) or 4 (22/37, 59%). Retention was unrelated to participant demographic characteristics, previous use of Woebot, psychiatric diagnoses, primary problematic substance, depressive symptoms, pain, cravings, confidence, substance use occasions, AUDIT-C scores, or DAST-10 scores (all *P* values>.102). Missing data on individual survey items was minimal. In a single instance, a participant’s average score values were imputed when missing 1 item on the PHQ-8. Participants were prompted to report craving and pain ratings within the W-SUDs app on a daily basis. The data were aggregated so that if participants provided multiple ratings within a day, the scores were averaged. To examine changes over time, generalized estimating equation linear models were run with week entered as a factor, setting week 1 as the reference category.

## Results

### Sample Characteristics

Table 1 presents the baseline characteristics of the participants. According to zip code, the sample was drawn from 31 US states, and at baseline, nearly all participants (99/101, 98.0%) reported sheltering in place during the COVID-19 pandemic. Most (73/101, 72.3%) reported a lifetime psychiatric diagnosis, most commonly generalized anxiety disorder (49/101, 48.5%) and unipolar depression (45/101, 44.6%), with 47.5% (48/101) reporting multiple lifetime psychiatric diagnoses; few (6/101, 5.9%) reported a SUD diagnosis, 43.6% (44/101) were currently taking psychiatric medication, and 25.7% (26/101) were currently in therapy.

**Table 1:** Sample characteristics at baseline (N=101).

Variable		Mean (SD); range	Value, n (%)
Age (years)		36.8 (10.0); 19-62	N/A^a^
**Sex**			
	Female	N/A	76 (75.2)
	Male	N/A	25 (24.8)
**Race and ethnicity**			
	Non-Hispanic White	N/A	79 (78.2)
	Hispanic/Latinx	N/A	4 (4.0)
	Non-Hispanic Black/African-American	N/A	4 (4.0)
	Non-Hispanic Asian-American	N/A	3 (3.0)
	Multiethnic	N/A	7 (6.9)
	Other or missing	N/A	4 (4.0)
**Marital status**			
	Married or cohabitating or partnered	N/A	54 (53.5)
	Divorced or separated or widowed	N/A	14 (13.9)
	Single or never married	N/A	33 (32.7)
**Employment status**			
	Employed full-time	N/A	62 (61.4)
	Employed part-time	N/A	11 (10.9)
	Unemployed, job-seeking	N/A	12 (11.9)
	Other (eg, retired, disabled, homemaker, and student)	N/A	16 (15.8)
**COVID-19 situation**			
	Sheltering in place, lockdown, quarantined	N/A	99 (98.0)
	No restrictions	N/A	2 (2.0)
**Lifetime psychiatric diagnoses**			
	Unipolar depression	N/A	45 (44.6)
	Bipolar or manic depression	N/A	10 (9.9)
	Anxiety disorder	N/A	49 (48.5)
	Posttraumatic stress disorder	N/A	19 (18.8)
	Attention deficit hyperactivity disorder	N/A	15 (14.9)
	Other (eg, obsessive compulsive disorder, eating disorder, and personality disorder)	N/A	12 (11.9)
	Substance use disorder	N/A	6 (5.9)
	Multiple psychiatric diagnoses	N/A	48 (47.5)
	No lifetime psychiatric diagnoses	N/A	28 (27.7)
**Therapy experience**			
	Never	N/A	30 (29.7)
	Formerly	N/A	45 (44.6)
	Currently	N/A	26 (25.7)
Currently taking psychiatric medication		N/A	44 (43.6)
**Patient Health Questionnaire-8 item depression (possible range 0-24)**		10.8 (5.8); 0-24	N/A
	10+ moderate-to-severe	N/A	54 (53.5)
**General Anxiety Disorder-7 item anxiety (possible range 0-21)**		9.6 (5.7); 0-21	N/A
	10+ moderate-to-severe	N/A	47 (46.5)
Pain intensity in the past 7 days (possible range 0-100)		20.4 (22.3); 0-80	N/A
**Pain interfere with normal work in the past 30 days**			
	Not at all	N/A	60 (59.4)
	A little bit	N/A	23 (22.8)
	Moderately	N/A	9 (8.9)
	Quite a bit	N/A	7 (6.9)
	Extremely	N/A	2 (2.0)
**Primary substance**			
	Alcohol	N/A	69 (68.3)
	Cannabis	N/A	20 (19.8)
	Stimulants or cocaine	N/A	7 (6.9)
	Other (eg, club drugs, pain killers, and sedatives)	N/A	5 (5.0)
Indicated multi-substances		N/A	37 (36.6)
**Past 30 days of substance use^b^ (days), mean (SD) and n (%) reporting any past 30-day use**			
	Alcohol	19.4 (9.2); 1-30	88 (87.1)
	Cannabis	19.4 (12.2); 1-30	50 (49.5)
	Sedatives	5.3 (5.3); 1-15	19 (18.8)
	Hallucinogens	2.0 (1.2); 1-5	10 (9.9)
	Prescription stimulants	21.9 (11.0); 2-30	10 (9.9)
	Cocaine	3.6 (4.0); 1-10	5 (5.0)
	Methamphetamine	22.5 (13.1); 3-30	4 (4.0)
	Inhalants	5.8 (3.3); 2-10	4 (4.0)
	Prescription opioids	12.4 (15.2); 1-30	5 (5.0)
	Street opioids	0 (0); 0	0 (0)
Number of substance use occasions in the past 30 days		31.8 (17.7); 0-76	N/A
**Alcohol Use Disorders Identification Test-Concise (possible range 0-12)**		5.5 (3.1); 0-12	N/A
	Men (% with score 4+, clinical range)	5.2 (3.2); 0-11	18 (72)
	Women (% with score 3+, clinical range)	5.5 (3.1); 0-12	59 (78)
**Drug Abuse Screening Test-10 item (possible range 0-10)**		3.0 (2.6); 0-8	N/A
	% with score 3+, clinical range	N/A	56 (55.4)
**Bothered by cravings in the past 7 days**			
	Not at all	N/A	7 (6.9)
	A little bit	N/A	31 (30.7)
	Moderately	N/A	33 (32.7)
	Quite a bit	N/A	23 (22.8)
	Extremely	N/A	7 (6.9)
**Current confidence scores^c^ (possible range 0%-100%)**			
	Negative emotional	38.5 (30.1); 0-100	N/A
	Negative physical	50.9 (33.4); 0-100	N/A
	Positive emotional	60.5 (31.7); 0-100	N/A
	Testing personal control	54.1 (34.2); 0-100	N/A
	Urges and temptations	41.1 (28.7); 0-100	N/A
	Interpersonal conflict	44.4 (30.9); 0-100	N/A
	Social pressure	49.2 (33.4); 0-100	N/A
	Positive social	46.0 (31.2); 0-100	N/A
	Overall confidence average score	48.1 (22.1); 0-100	N/A

^a^N/A: not applicable.

^b^Six participants reported no substance use in the past 30 days at baseline. The mean days of use were calculated among those who reported any use of that substance in the past 30 days.

^c^Values presented are percentages.

### Substance Use at Pretreatment

Self-identified primary problematic substances were alcohol (69/101, 68.3%), cannabis (20/101, 19.8%), stimulants or cocaine (7/101, 6.9%), and other (5/101, 4.9%). Over a third (37/101, 36.6%) indicated problems with multiple substances. Most (88/101, 87.1%) reported use of alcohol in the past month; among past month drinkers, alcohol use averaged 19.4 of the past 30 days. About half (50/101, 49.5%) reported use of cannabis in the past month, among users averaging 19.4 of the past 30 days. Less common was use of sedatives (19/101, 18.8%), hallucinogens (10/101, 9.9%), and prescription stimulants (10/101, 9.9%) in the past month. None of the participants reported use of street opioids in the past month. Combining reported days of use across substances, the number of use occasions in the past 30 days averaged 31.8 (SD 17.7) with a wide range of 0-76. At baseline, AUDIT-C scores averaged 5.5 (SD 3.1) for the overall sample, with 72% (18/25) of men and 78% (59/76) of women scoring in the clinical range. DAST-10 scores averaged 3.0 (SD 2.6), with 55.4% (56/101) scoring in the clinical range. Nearly two-thirds (63/101, 62.4%) of the sample reported being bothered in the past 7 days by moderate-to-extreme cravings or urges to drink alcohol or use drugs. Participants’ confidence in 8 domains to resist urges to use substances ranged from an average of 60.5% (SD 31.7) for positive emotional states to 38.5% (SD 30.1) for negative emotional states, with an overall average of 48.1% (SD 22.1) and a wide range of 1%-100%.

### W-SUDs Use and Within-App User Feedback

Among the full sample (N=101), for the 8-week treatment period, participants’ use of W-SUDs averaged 15.7 days (SD 14.2; median 10; IQR 20) or 2.0 times per week, with an average of 600.7 user sent messages (SD 556.5; median 360; IQR 763) or 75.1 messages per week and engagement on average with 12.1 modules (SD 8.3; median 9; IQR 12.5), which consist of psychoeducational lessons and psychotherapeutic tools for mood and behavior change. An indicator of intervention engagement over time, Multimedia Appendix 1 shows the percentage of participants actively sending messages by treatment week and, among those participating each week, their average number of messages. The types of conversations vary in length; therefore, the total number of messages sent does not necessarily reflect the richness of content reviewed. In addition, the individuals in each week are not necessarily the same across weeks. For example, someone could have sent messages in weeks 2 to 4 and 6 to 7 but not in weeks 5 or 8. The sample completed an average of 7.9 psychoeducational lessons (SD 7.6; median 4; IQR 12). Lesson completion rates were highest (>50%) for content concerning COVID-19, urge surfing, and SUD labels and lowest (<5%) for content concerning sleep and grief. Lesson acceptability ratings were high across the board, with 94.0% (562/598) of completed lessons receiving thumbs up. Participants used an average of 4.3 tools (SD 1.4; median 4; IQR 1). Mood impact after tool utilization, denoting in-vivo mood modulation, was predominately positive (better=70%, same=24%, and worse=6%). In total, 14 of the 101 users (13.9%) completed all of the psychoeducational lessons in W-SUDs before the end of the 8-week intervention period.

### W-SUDs Mood, Craving, and Pain Ratings

A total of 1571 mood ratings were entered into the W-SUDs app by 90 of the 101 (89.1%) participants, with each participant entering on average 17.5 mood ratings (SD 16.1; median 10; IQR 25.3) or 2.2 per week. A total of 1399 craving and 1403 pain ratings were entered into the W-SUDs app by 87 of the 101 participants (86.1%), with each participant providing an average of 16.1 ratings (SD 14.8; median 9; IQR 21) for cravings and 16.1 ratings (SD 14.9; median 9; IQR 21) for pain. Table 2 shows the number of participants providing craving ratings for each week and summarizes the generalized estimating equation model analyzing craving ratings over time. Compared with week 1, craving ratings were significantly lower at weeks 4 through 9. By weeks 8 and 9, craving ratings were reduced by approximately half of the sample’s mean rating at week 1. In contrast, pain ratings did not differ significantly by week and over the 9 weeks averaged 2.3 (SD 2.1), on a scale of 0 to 10.

**Table 2:** Participants’ (N=101) craving ratings from week 1 to week 9 reported in the Woebot for the treatment of substance use disorders (W-SUDs) app.

Variable	Value, n^a^ (%)	Craving, mean (SD)^b^	β	SE	Wald *X*^2^ (*df*)	*P* value	Exp (B)	95% CI
Week 1^c^	82 (81.2)	1.59 (0.11)	0^d^	Ref	Ref	Ref	Ref	Ref
Week 2	69 (68.3)	1.48 (0.12)	−.11	0.09	1.35 (1)	.25	0.90	0.761.08
Week 3	55 (54.5)	1.32 (0.14)	−.27	0.14	3.59 (1)	.06	0.77	0.58-1.01
Week 4	48 (47.5)	1.21 (0.17)	−.38	0.16	5.33 (1)	.02	0.69	0.50-0.95
Week 5	39 (38.6)	0.88 (0.15)	−.71	0.16	21.21 (1)	<.001	0.49	0.36-0.66
Week 6	32 (31.7)	1.01 (0.21)	−.58	0.20	8.46 (1)	.004	0.56	0.38-0.83
Week 7	30 (29.7)	0.98 (0.18)	−.61	0.20	9.19 (1)	.002	0.54	0.37-0.81
Week 8	24 (23.8)	0.81 (0.19)	−.78	0.21	13.84 (1)	<.001	0.46	0.30-0.69
Week 9^e^	20 (19.8)	0.86 (0.2)	−.73	0.21	11.62 (1)	.001	0.48	0.32-0.73

^a^Number of participants reporting their craving at least once each week with response options of not at all (0), a little bit (1), moderately (2), quite a bit (3), or extremely (4).

^b^Model estimated marginal means (SD).

^c^Week 1 is the reference group to which all other weeks are compared.

^d^Set to zero as the reference category.

^e^Woebot for the treatment of substance use disorders is offered as an 8-week treatment; however, participants could continue to use the app.

### Changes Pre- to Posttreatment

Table 3 shows scores for the participants who completed assessments at both pre- and posttreatment. In paired sample *t* tests, confidence scores overall and in all 8 domains significantly increased from pre- to posttreatment (all *P* values<.05). In addition, significant reductions were observed from pre- to posttreatment in past month substance use occasions, AUDIT-C and DAST-10 scores (overall and among those in the clinical range at pretreatment), and PHQ-8 depression and GAD-7 anxiety scores (all *P* values<.05). A McNemar test indicated significant reductions in cravings, with more participants reporting little to no cravings and fewer reporting moderate-to-extreme cravings from pre- to posttreatment (*P*<.001). Reports of pain intensity and pain interference with work did not change significantly from pre- to posttreatment.

A greater decline in the AUDIT-C score was associated with greater reductions in use occasions (*r*=0.48), PHQ-8 depression (*r*=0.36), and GAD-7 anxiety (*r*=0.34) scores and with increases in confidence (*r*=−0.39; all *P* values<.02). A greater decline in the DAST-10 score was associated with greater reductions in PHQ-8 depression (*r*=0.40; *P*<.01) but not with the number of use occasions (*r*=0.10), confidence (*r*=−0.12), or GAD-7 anxiety (*r*=0.21).

Of the 14 *t* tests, only 1 was statistically significant as to whether participants currently in therapy or taking psychiatric medications showed greater pre- to posttreatment changes in substance use (use occasions, AUDIT-C, and DAST-10), confidence, mood (PHQ-8 and GAD-7), or pain. The finding was that participants currently in therapy reported greater reductions from pre- to posttreatment in depressive symptoms (n=16; mean change −4.7, SD 4.5) than those not currently in therapy (n=35; mean change −0.9, SD 5.1; t_49_=2.55; *P*=.01).

**Table 3:** Pre- to posttreatment changes in substance use and mental health measures (n=51).

Variable		Pretreatment	Posttreatment	*t* test (*df*)	*P* value
Substance use occasions^a^, mean (SD)		29.5 (14.0)	20.1 (17.8)	−4.72 (50)	<.001
**Alcohol Use Disorders Identification Test-Concise, mean (SD)**					
	Full sample	5.3 (2.9)	4.0 (3.2)	−3.58 (50)	<.001
	At-risk at pretreatment^b^	6.7 (2.0)	4.9 (3.2)	−3.92 (38)	<.001
**Drug Abuse Screening Test-10 item, mean (SD)**					
	Full sample	2.9 (2.7)	1.7 (2.4)	−4.25 (50)	<.001
	At-risk at pretreatment^b^	5.3 (1.5)	3.1 (2.6)	−5.00 (26)	<.001
**Confidence scores (0%-100%), mean (SD)**					
	Negative emotional	37.2 (28.4)	56.3 (27.9)	3.86 (50)	<.001
	Negative physical	49.2 (31.1)	64.8 (30.7)	3.41 (50)	.001
	Positive emotional	61.7 (28.2)	75.2 (26.8)	2.98 (50)	.004
	Testing personal control	51.7 (31.0)	63.4 (30.6)	2.42 (50)	.03
	Urges and temptations	37.9 (23.6)	57.5 (28.6)	5.52 (50)	<.001
	Interpersonal conflict	40.9 (28.2)	61.2 (31.1)	4.62 (50)	<.001
	Social pressure	43.8 (32.5)	63.4 (33.7)	3.64 (50)	.001
	Positive social	45.5 (31.6)	60.8 (30.2)	3.56 (50)	.001
	Overall confidence average score	46.0 (19.3)	62.8 (22.4)	5.62 (50)	<.001
**Bothered by cravings in the past 7 days, n (%)**					
	Not at all or a little bit	16 (31.4)	27 (53)	N/A^c^	.013^d^
	Moderately or quite a bit or extremely	35 (68.6)	24 (47)	N/A	.013^d^
Pain intensity in the past 7 days, mean (SD)		24.3 (22.2)	24.2 (22.0)	−0.02 (50)	.982
**Pain interfere with work in the past 30 days, n (%)**					
	Not at all or a little bit	40 (78.4)	40 (78)	N/A	1.00^d^
	Moderately or quite a bit or extremely	11 (21.5)	11 (22)	N/A	1.00^d^
Patient Health Questionnaire-8 item depression, mean (SD)		10.7 (5.3)	8.6 (5.1)	−2.91 (50)	.005
General Anxiety Disorder-7 item anxiety, mean (SD)		10.1 (5.7)	7.8 (5.3)	−3.45 (50)	.001

^a^Reflects number of days of use summed across substances.

^b^Analyses run for the subgroup of participants scoring in the clinical range at pretreatment, which are scores of 4+ for men and 3+ for women on the Alcohol Use Disorders Identification Test-Concise and scores of 3+ on the Drug Abuse Screening Test-10 item.

^c^N/A: not applicable.

^d^*P* value obtained with McNemar’s test.

### Serious Adverse Events

Among the 51 participants who completed the posttreatment assessment, 1 reported a serious adverse event. An individual reported hospitalization for treatment of sepsis secondary to switching from smoking to injecting illicit drugs, shortly before or at the start of study participation, and was deemed by the DSMB to be unrelated to study involvement.

### Feasibility and Acceptability Ratings

Table 4 shows the mean scores and ranges of the 4 feasibility and acceptability measures completed posttreatment. On the individual CSQ-8 items, the majority (35/51, 69%) indicated that they would return to the program, reported that interactions with W-SUDs helped them deal more effectively with their problems (35/51, 69%), were mostly or very satisfied overall (36/51, 71%), were satisfied with the amount of help received (37/51, 73%), rated the quality of interaction on W-SUDs as good or excellent (39/51, 76%), would recommend W-SUDs to a friend (39/51, 76%), and received the kind of service they wanted (41/51, 80%). A lower percentage of participants stated that W-SUDs met most or all of their needs (22/51, 43%). Scores for the 3 WAI-SR subscales, with identical response options, differed significantly from each other in pairwise *t* test comparisons (all *P* values<.05), with the highest ratings on development of an affective bond to Woebot, followed by agreement on the tasks of treatment and then agreement on the goals of treatment.

**Table 4:** Woebot for the treatment of substance use disorders (W-SUDs) posttreatment feasibility and acceptability ratings (n=51).

Measure		Mean (SD); range
**Usage Rating Profile-Intervention (12 items, possible range 12-72)**		
	Feasibility (6 items, range 6-36)	28.5 (5.7); 11-36
	Acceptability (6 items, range 6-36)	25.6 (7.3); 6-36
Client Satisfaction Questionnaire (8 items, possible range 8-32)		23.2 (5.5); 8-31
**Working Alliance Inventory-Short Revised (12 items, possible range 12-60)**		40.8 (12.5); 12-60
	Goal agreement (4 items, range 4-20)	12.4 (4.4); 4-20
	Task agreement (4 items, range 4-20)	13.0 (4.8); 4-20
	Affective bond formation (4 items, range 4-20)	15.4 (4.2); 4-20

CSQ-8 satisfaction scores did not differ by any measured participant characteristics, including sex, race or ethnicity, marital and employment status, age, primary substance of abuse, or history of a psychiatric diagnosis. CSQ-8 satisfaction scores also did not differ by baseline measures of depression, anxiety, pain, craving, confidence, substance use occasions, AUDIT-C, or DAST-10 scores. Non-Hispanic White participants had higher URP-I-Acceptability ratings (*F*_1,50_=8.32; *P*=.006) and higher WAI-SR scores (*F*_1,50_=5.08; *P*=.03) than participants from other racial or ethnic groups. In addition, URP-I-Acceptability ratings were higher among participants who reported moderate-to-extreme craving at baseline (*F*_1,50_=5.21; *P*=.03). Finally, older age (*r*=0.36; *P*=.01) and reporting of moderate-to-extreme impairment due to pain at baseline (*F*_1,50_=4.36; *P*=.04) were associated with higher URP-I-Feasibility ratings.

A greater reduction in substance use occasions from pre- to posttreatment was significantly associated with higher WAI-SR (*r*=−0.37; *P*=.008) and URP-I-Acceptability (*r*=−0.30; *P*=.03) scores. An increase in confidence to resist urges to use substances was also associated with higher scores on the WAI-SR (*r*=0.30; *P*=.03), URP-I-Acceptability (*r*=0.33; *P*=.02), and CSQ-8 (*r*=0.28; *P*=.045). Changes in AUDIT-C, DAST-10, depression, and anxiety measures were not associated with acceptability and feasibility ratings.

## Discussion

### Principal Findings

W-SUDs, an automated conversational agent, was feasible to deliver, engaging, and acceptable and was associated with significant improvements pre- to posttreatment in self-reported measures of substance use, confidence, craving, depression, and anxiety and in-app measures of craving. The W-SUDs app registration rate among those who completed the baseline survey was 78.9% (101/128), comparable with other successful mobile health interventions [47]. As expected, the use of the W-SUDs app was highest early in treatment and declined over the 8 weeks. Study of engagement with digital health apps has been growing, with no consensus yet on ideal construct definitions [48-50]. Simply reporting the number of messages or minutes spent on an app over time may undermine clarity and genuine understanding of the type and manifestation of app utilization related to clinical outcomes of interest [51]. Further research in this area is warranted.

The observed reductions from pre- to posttreatment measures of depression and anxiety symptoms were consistent with a previous evaluation of Woebot conducted with college students self-identified as having symptoms of anxiety and depression [18]. Furthermore, in this study, treatment-related reductions in depression and anxiety symptoms were associated with declines in problematic substance use. Declines in depressive symptoms observed from pre- to posttreatment were greater among the participants in therapy.

This study also examined working alliance, proposed to mediate clinical outcomes in traditional therapeutic settings [52]. Traditionally, working alliance has been characterized as the cooperation and collaboration in the therapeutic relationship between the patient and the therapist [53-55]. The role of working alliance in relationally based systems and digital therapeutics has been previously considered [16,17,56]; the potential of alliance to mediate outcomes in Woebot should be further validated in future studies adequately powered to examine mediators of change.

Measures of physical pain did not change with the use of W-SUDs as reported in pre- and posttreatment measures or within the app; however, the sample’s baseline ratings of pain intensity and pain interference were low. Although not a direct intervention target, pain was measured due to the potential for use of substances to self-treat physical pain and the possibility that pain may worsen if substance use was reduced, which was not observed here.

Within-app lesson completion and content acceptability were high for the overall sample, although there was a wide range of use patterns. Most participants used all facets of the W-SUDs app: tracked their mood, cravings, and pain; completed on average over 7 psychoeducational lessons; and used tools in the W-SUDs app. Only about half of the sample completed the posttreatment assessment, with better retention among those screening higher on the CAGE-AID. That is, those with more severe substance use problems at the start of the study, and hence in greater need of the intervention, were more likely to complete the posttreatment evaluation. None of the other measured variables distinguished those who did and did not complete the posttreatment evaluation. This level of attrition is commensurate with other digital mental health solution trial attrition rates [47,57].

### Comparison With Previous Work

By addressing problematic substance use, including but not limited to alcohol, the W-SUDs intervention supports and extends a growing body of literature on the use of automated conversational agents (or chatbots) and other mobile apps to support behavioral health. A systematic review of mobile and web-based interventions targeting the reduction of problematic substance use found that most web-based interventions produced significant short-term improvements in at least one measure of problematic substance use [6]. Mobile apps were less common than web-based interventions, with weaker evidence of efficacy and some indication of causing harm (ie, inadvertently helping users increase, rather than decrease, their blood alcohol level while partying). However, mobile interventions can be efficacious. Electronic screening and brief intervention programs, which use mobile tools to screen for excessive alcohol use and deliver personalized feedback, have been found to effectively reduce alcohol consumption and alcohol-related problems [58]. However, rigorous evaluation trials of digital interventions targeting nonalcohol substance use are limited [7]. Furthermore, although a systematic review concluded that conversational agents showed preliminary efficacy in reducing psychological distress among adults with mental health concerns compared with inactive control conditions [27], this is the first published study of a conversational agent adapted for substance use.

### Study Strengths

Study strengths include study enrollment being double the initial recruitment goal, reflecting interest in W-SUDs. Most participants reported lifetime psychiatric diagnoses, and approximately half of the participants endorsed current moderate-to-severe levels of depression or anxiety. W-SUDs was used on average twice per week during the 8-week program. From pre- to posttreatment with W-SUDs, participants reported significant improvements in multiple measures of substance use and mood. The delivery modality of W-SUDs offered easy, immediate, and stigma-free access to emotional support and substance use recovery information, particularly relevant during a time of global physical distancing and sheltering in place. More time spent at home, coupled with reduced access to in-person mental health care, may have increased enrollment and engagement with the app. Although further data on recruitment and enrollment are warranted, these early findings suggest that individuals with SUDs are indeed interested in obtaining support for this condition from a fully digitalized conversational agent.

### Limitations and Future Directions

This study had a single-group design, and the outcomes were short term and limited to posttreatment, thus limiting the strength of inferences that can be drawn. The sample was predominately female and identified as non-Hispanic White, and the majority were employed full-time. Non-Hispanic White participants reported higher program acceptability on 2 of the 4 measures compared with participants from other racial or ethnic groups. Future research on W-SUDs will use a randomized design, with longer follow-up, and focus on recruitment of a more diverse population to better inform racial or ethnic cultural programmatic tailoring, using quotas to ensure racial or ethnic diversity in sampling. Notably, although recruited from across the United States, nearly all participants (99/101, 98.0%) were sheltering in place at the time of study enrollment due to the COVID-19 pandemic, which may have affected substance use patterns and mood as well as interest in a digital health intervention. Notably, however, alcohol sales in the United States increased during the COVID-19 pandemic [59]. The primary outcomes of substance use, cravings, confidence, mood, and program acceptability were standard measures with demonstrated validity and reliability. The limitations were that all were self-reported, and acceptability measures were not open-ended or qualitative. Few participants were misusing opioids, likely due to study exclusion designed to mitigate risk, namely, the requirement of engagement with medication-assisted treatment and no history of opioid overdose requiring Narcan (naloxone). Notably, nearly 1400 people with interest in a program for those with substance use concerns were excluded due to low severity on the CAGE-AID screener. Worth testing is the utility of digital health programs for early intervention on substance misuse that is subsyndromal.

Building upon the findings of this study, future research will evaluate W-SUDs in a randomized controlled trial with a more racially or ethnically diverse sample, balanced on sex and primary problematic substance of use; will employ greater strategies for study retention (eg, increased incentives, obtaining phone contact details, and sending more outreach reminders); and will be conducted during a period with less restrictions on social contacts and physical mobility. Randomized controlled evaluations of conversational agent interventions relative to other treatment modalities are required [27,60].

### Conclusions

This study is the first empirical evaluation of an SUD-focused digital therapeutic delivered via a fully automated conversational agent. The therapeutic approach is acceptable, feasible, and safe. The study observed significant reductions in substance use and cravings in the context of population-level shifts in the pattern of substance use during a global pandemic. The scalability and accessibility of an automated program coupled with the growing problem of substance use suggest the potential for an engaging and effective therapeutic to reduce the burden of SUDs. Further research is needed to quantify the adoption potential and population impacts of an efficacious digital therapeutic conversational agent for SUD treatment.

## Appendix

Multimedia Appendix 1 Percentage of participants sending messages to Woebot for the treatment of substance use disorders and the average number of messages sent by them each week. W-SUDs: Woebot for the treatment of substance use disorders.

## Abbreviations

AUDIT-C: Alcohol Use Disorders Identification Test-Concise
CAGE-AID: Cut down, Annoyed, Guilty, Eye opener-Adapted to Include Drugs
CBT: cognitive behavioral therapy
CSQ-8: Client Satisfaction Questionnaire-8 questions
DAST-10: Drug Abuse Screening Test-10
DSMB: Data Safety Monitoring Board
GAD-7: General Anxiety Disorder-7 item
PHQ-8: Patient Health Questionnaire-8 item
SUD: substance use disorder
URP-I: Usage Rating Profile-Intervention
WAI-SR: Working Alliance Inventory-Short Revised
W-SUDs: Woebot for the treatment of substance use disorders

## References

[1] World Health Organization (2018). Global status report on alcohol and health.

[2] (2017). Institute for health metrics and evaluation. Global burden of disease study 2016 results database.

[3] Substance Abuse and Mental Health Services Administration (2019). Key substance use and mental health indicators in the United States: results from the 2018 National Survey on Drug Use and Health. Center for Behavioral Health Statistics and Quality, Substance Abuse and Mental Health Services Administration.

[4] Grant BF, Stinson FS, Dawson DA, Chou SP, Dufour MC, Compton W, Pickering RP, Kaplan K (2004). Prevalence and co-occurrence of substance use disorders and independent mood and anxiety disorders: results from the National Epidemiologic Survey on alcohol and related conditions. Arch Gen Psychiatry.

[5] Keane H (2018). Facing addiction in America: The Surgeon General's Report on Alcohol, Drugs, and Health U.S. Department of Health and Human Services, Office of the Surgeon General Washington, DC, USA: U.S. Department of Health and Human Services, 2016 382 pp. online (grey literature): https://addiction.surgeongeneral.gov/. Drug Alcohol Rev.

[6] Giroux I, Goulet A, Mercier J, Jacques C, Bouchard S (2017). Online and mobile interventions for problem gambling, alcohol, and drugs: a systematic review. Front Psychol.

[7] Boumparis N, Schulte MHJ, Riper H (2019). Digital mental health for alcohol and substance use disorders. Curr Treat Options Psych.

[8] Perrin A, Turner E (2019). Smartphones help blacks, Hispanics bridge some - but not all - digital gaps with whites. Pew Research Center.

[9] (2019). Mobile fact sheet. Pew Research Center.

[10] Krebs P, Duncan DT (2015). Health app use among US mobile phone owners: a national survey. JMIR Mhealth Uhealth.

[11] Smith A (2015). U.S. smartphone use in 2015. Pew Research Center.

[12] Vaidyam AN, Wisniewski H, Halamka JD, Kashavan MS, Torous JB (2019). Chatbots and conversational agents in mental health: a review of the psychiatric landscape. Can J Psychiatry.

[13] Baumel A, Muench F, Edan S, Kane JM (2019). Objective user engagement with mental health apps: systematic search and panel-based usage analysis. J Med Internet Res.

[14] Torous J, Nicholas J, Larsen ME, Firth J, Christensen H (2018). Clinical review of user engagement with mental health smartphone apps: evidence, theory and improvements. Evid Based Ment Health.

[15] Lucas GM, Gratch J, King A, Morency L (2014). It’s only a computer: Virtual humans increase willingness to disclose. Computers in Human Behavior.

[16] Cook JE, Doyle C (2002). Working alliance in online therapy as compared to face-to-face therapy: preliminary results. Cyberpsychol Behav.

[17] Berry K, Salter A, Morris R, James S, Bucci S (2018). Assessing therapeutic alliance in the context of mHealth interventions for mental health problems: development of the mobile agnew relationship measure (mARM) questionnaire. J Med Internet Res.

[18] Fitzpatrick KK, Darcy A, Vierhile M (2017). Delivering cognitive behavior therapy to young adults with symptoms of depression and anxiety using a fully automated conversational agent (Woebot): a randomized controlled trial. JMIR Ment Health.

[19] Ly KH, Ly AM, Andersson G (2017). A fully automated conversational agent for promoting mental well-being: A pilot RCT using mixed methods. Internet Interv.

[20] Tielman ML, Neerincx MA, Bidarra R, Kybartas B, Brinkman W (2017). A therapy system for post-traumatic stress disorder using a virtual agent and virtual storytelling to reconstruct traumatic memories. J Med Syst.

[21] Gardiner PM, McCue KD, Negash LM, Cheng T, White LF, Yinusa-Nyahkoon L, Jack BW, Bickmore TW (2017). Engaging women with an embodied conversational agent to deliver mindfulness and lifestyle recommendations: a feasibility randomized control trial. Patient Educ Couns.

[22] Bickmore TW, Puskar K, Schlenk EA, Pfeifer LM, Sereika SM (2010). Maintaining reality: relational agents for antipsychotic medication adherence. Interact Comput.

[23] Bickmore TW, Mitchell SE, Jack BW, Paasche-Orlow MK, Pfeifer LM, Odonnell J (2010). Response to a relational agent by hospital patients with depressive symptoms. Interact Comput.

[24] Lucas GM, Rizzo A, Gratch J, Scherer S, Stratou G, Boberg J, Morency L (2017). Reporting mental health symptoms: breaking down barriers to care with virtual human interviewers. Front Robot AI.

[25] Philip P, Micoulaud-Franchi J, Sagaspe P, Sevin ED, Olive J, Bioulac S, Sauteraud A (2017). Virtual human as a new diagnostic tool, a proof of concept study in the field of major depressive disorders. Sci Rep.

[26] Tielman ML, Neerincx MA, van Meggelen M, Franken I, Brinkman W (2017). How should a virtual agent present psychoeducation? Influence of verbal and textual presentation on adherence. Technol Health Care.

[27] Gaffney H, Mansell W, Tai S (2019). Conversational agents in the treatment of mental health problems: mixed-method systematic review. JMIR Ment Health.

[28] (2004). NIAAA council approves definition of binge drinking. NIAAA Newsletter.

[29] Miner AS, Shah N, Bullock KD, Arnow BA, Bailenson J, Hancock J (2019). Key considerations for incorporating conversational AI in psychotherapy. Front Psychiatry.

[30] Brown RL, Rounds LA (1995). Conjoint screening questionnaires for alcohol and other drug abuse: criterion validity in a primary care practice. Wis Med J.

[31] Williams C, Wilson P, Morrison J, McMahon A, Walker A, Andrew W, Allan L, McConnachie A, McNeill Y, Tansey L (2013). Guided self-help cognitive behavioural therapy for depression in primary care: a randomised controlled trial. PLoS One.

[32] Salomonsson S, Santoft F, Lindsäter E, Ejeby K, Ingvar M, Öst LG, Lekander M, Ljótsson B, Hedman-Lagerlöf E (2020). Predictors of outcome in guided self-help cognitive behavioural therapy for common mental disorders in primary care. Cogn Behav Ther.

[33] Cully JA, Teten AL (2008). A therapist's guide to brief cognitive behavioral therapy. Department of Veterans Affairs, South Central Mental Illness Research, Education, and Clinical Center (MIRECC).

[34] Barth J, Munder T, Gerger H, Nüesch E, Trelle S, Znoj H, Jüni P, Cuijpers P (2013). Comparative efficacy of seven psychotherapeutic interventions for patients with depression: a network meta-analysis. PLoS Med.

[35] (2011). Appendix E: Glossary. National Institute for Health and Care Excellence; 2011. National Institute for Health and Care Excellence.

[36] Miller WR (2005). Are alcoholism treatments effective? The Project MATCH data: response. BMC Public Health.

[37] DiClemente CC, Corno CM, Graydon MM, Wiprovnick AE, Knoblach DJ (2017). Motivational interviewing, enhancement, and brief interventions over the last decade: a review of reviews of efficacy and effectiveness. Psychol Addict Behav.

[38] Brorson HH, Arnevik AE, Rand-Hendriksen K, Duckert F (2013). Drop-out from addiction treatment: a systematic review of risk factors. Clin Psychol Rev.

[39] Bush K, Kivlahan DR, McDonell MB, Fihn SD, Bradley KA (1998). The AUDIT alcohol consumption questions (AUDIT-C): an effective brief screening test for problem drinking. Ambulatory Care Quality Improvement Project (ACQUIP). Alcohol Use Disorders Identification Test. Arch Intern Med.

[40] Skinner HA (1982). The drug abuse screening test. Addictive Behaviors.

[41] Breslin FC, Sobell LC, Sobell MB, Agrawal S (2000). A comparison of a brief and long version of the situational confidence questionnaire. Behav Res Ther.

[42] Kroenke K, Strine TW, Spitzer RL, Williams JBW, Berry JT, Mokdad AH (2009). The PHQ-8 as a measure of current depression in the general population. J Affect Disord.

[43] Spitzer RL, Kroenke K, Williams JBW, Löwe B (2006). A brief measure for assessing generalized anxiety disorder: the GAD-7. Arch Intern Med.

[44] Briesch AM, Chafouleas SM, Neugebauer SR, Riley-Tillman TC (2013). Assessing influences on intervention implementation: revision of the usage rating profile-intervention. J Sch Psychol.

[45] Larsen DL, Attkisson CC, Hargreaves WA, Nguyen TD (1979). Assessment of client/patient satisfaction: development of a general scale. Eval Prog Plan.

[46] Hatcher RL, Gillaspy JA (2006). Development and validation of a revised short version of the working alliance inventory. Psychoth Res.

[47] Cliffe B, Croker A, Denne M, Smith J, Stallard P (2020). Digital cognitive behavioral therapy for insomnia for adolescents with mental health problems: feasibility open trial. JMIR Ment Health.

[48] O'Brien H (2016). Theoretical perspectives on user engagement. Why Engagement Matters.

[49] Perski O, Blandford A, West R, Michie S (2017). Conceptualising engagement with digital behaviour change interventions: a systematic review using principles from critical interpretive synthesis. Transl Behav Med.

[50] Holdener M, Gut A, Angerer A (2020). Applicability of the user engagement scale to mobile health: a survey-based quantitative study. JMIR Mhealth Uhealth.

[51] Chien I, Enrique A, Palacios J, Regan T, Keegan D, Carter D, Tschiatschek S, Nori A, Thieme A, Richards D, Doherty G, Belgrave D (2020). A machine learning approach to understanding patterns of engagement with internet-delivered mental health interventions. JAMA Netw Open.

[52] Safran JD, Muran JC (1996). The resolution of ruptures in the therapeutic alliance. J Consult Clin Psychol.

[53] Bordin ES (1979). The generalizability of the psychoanalytic concept of the working alliance. Psychotherapy: Theory, Research & Practice.

[54] Horvath AO, Symonds BD (1991). Relation between working alliance and outcome in psychotherapy: a meta-analysis. J Counsel Psychol.

[55] Ruwaard J, Schrieken B, Schrijver M, Broeksteeg J, Dekker J, Vermeulen H, Lange A (2009). Standardized web-based cognitive behavioural therapy of mild to moderate depression: a randomized controlled trial with a long-term follow-up. Cogn Behav Ther.

[56] Bickmore T, Gruber A, Picard R (2005). Establishing the computer-patient working alliance in automated health behavior change interventions. Patient Educ Couns.

[57] Pratap A, Neto EC, Snyder P, Stepnowsky C, Elhadad N, Grant D, Mohebbi MH, Mooney S, Suver C, Wilbanks J, Mangravite L, Heagerty PJ, Areán P, Omberg L (2020). Indicators of retention in remote digital health studies: a cross-study evaluation of 100,000 participants. NPJ Digit Med.

[58] (2012). Preventing excessive alcohol consumption: electronic Screening and Brief Intervention (e-SBI). Community Preventive Services Task Force.

[59] Rebalancing the ‘COVID-19 effect’ on alcohol sales.

[60] Laranjo L, Dunn AG, Tong HL, Kocaballi AB, Chen J, Bashir R, Surian D, Gallego B, Magrabi F, Lau AYS, Coiera E (2018). Conversational agents in healthcare: a systematic review. J Am Med Inform Assoc.

