# Supplementary figures and images for: A Therapeutic Relational Agent for Reducing Problematic Substance Use (Woebot): Development and Usability Study
Source: J Med Internet Res. 2021 Mar 23;23(3):e24850. doi: 10.2196/24850 (PMC8074987; doi:10.2196/24850)

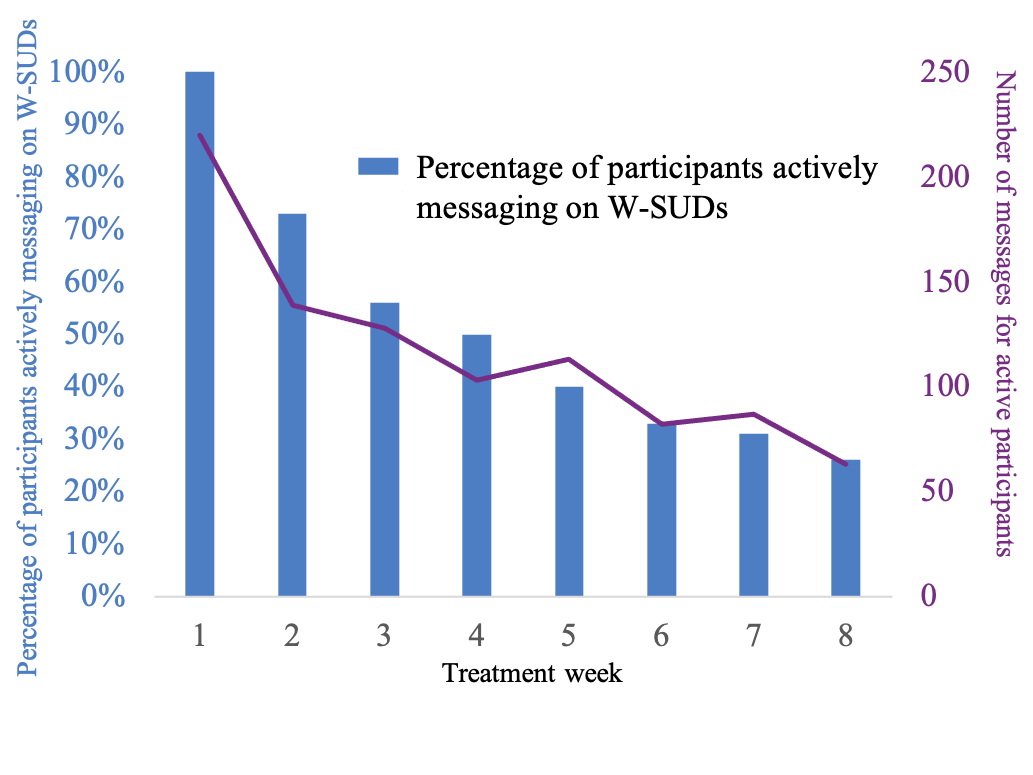

Supplement: Multimedia Appendix 1 [file jmir_v23i3e24850_app1.png]
